# Intratumoural injection of the toll-like receptor-2/6 agonist ‘macrophage-activating lipopeptide-2’ in patients with pancreatic carcinoma: a phase I/II trial

**DOI:** 10.1038/sj.bjc.6603903

**Published:** 2007-07-31

**Authors:** J Schmidt, T Welsch, D Jäger, P F Mühlradt, M W Büchler, A Märten

**Affiliations:** 1 Department of Surgery, University of Heidelberg, Heidelberg 69120, Germany; 2 National Centre for Tumour Diseases, University of Heidelberg, Heidelberg 69120, Germany; 3 Wound Healing Research Group, BioTec Gründerzentrum, Braunschweig 38124, Germany

**Keywords:** pancreatic adenocarcinoma, toll-like receptor, immunotherapy

## Abstract

This phase I/II trial examined safety and efficacy of the toll-like receptor 2/6 agonist MALP-2 in combination with gemcitabine in patients with incompletely resectable pancreas carcinomas. MALP-2 is a toll-like receptor 2/6 agonist, acts as an immunological adjuvant, and has been described recently to prolong survival in a mouse model of an orthotopic, syngeneic pancreas tumour. Male and female patients with incompletely resectable pancreas carcinomas were eligible while those with R0 or R1 resections or with peritoneal carcinosis were excluded. Ten patients were injected intratumourally during surgery with 20–30 *μ*g MALP-2 followed by postoperative chemotherapy. Samples were taken from peripheral blood and wound secretion, and assayed for cell content, cytokine and CRP levels, and NK activity. An MALP-2 dose of 20 *μ*g was well tolerated. Clear signs of local MALP-2 effects were presented by the influx of lymphocytes and monocytes in wound secretions, and abolishment of inhibition of NK activity. The actual mean survival is 17.1±4.2 months; the median survival being 9.3 months. Two patients are still alive after 31 months. Up to 20 *μ*g MALP-2 was well tolerated, and no systemic side effects were noted. The mean survival of 17.1 months is remarkably high.

Carcinoma of the exocrine pancreas has an extremely poor prognosis. The 5-year survival rate is <1% with a median survival of 4–6 months. Even with radical curative surgery and adjuvant chemotherapy (CT) the 5-year survival is at best 25% in specialised centres (Neoptolemos *et al*, 2001). After palliative surgical intervention, the 1-year survival is 20% and the 5-year survival rate is 3% (Koslowsky *et al*, 2001; Raraty *et al*, 2002). The standard palliative treatment with gemcitabine results in a median survival of 5.7 months (Burris *et al*, 1997). Recently, data from a phase III trial showed that treatment with gemcitabine plus capecitabine increased survival to 7.4 months (Cunningham *et al*, 2005).

Intraoperative radiotherapy (IORT) is another therapeutic strategy in patients with locally advanced disease. It involves delivery of high doses of irradiation to the pancreas and to the surgical bed following pancreatic resection, while uninvolved and dose-limiting tissues are displaced. Ihse *et al* (2005) reported a median survival of 7 months from 37 patients with advanced pancreatic carcinoma.O'Connor *et al* (2005) reported a median survival of 11 months for 24 patients who underwent surgical bypass and IORT.

Immunotherapy could be another strategy for cancer treatment. General barriers to successful tumour vaccination consist of various escape mechanisms developed by tumour cells to avoid immune attack. Malignant cells evade immunosuppression by downregulating intrinsic immunogenicity (Bissell and Radisky, 2001), or by induction of tolerance or anergy, in particular of dendritic cells (DC) (Wittke *et al*, 1999). Jaffee *et al*(1998) showed in preclinical studies with murine tumour models that tumour cell vaccines engineered to secrete GM-CSF in a paracrine fashion elicit systemic immune responses capable of eliminating small amounts of established pancreatic tumour. Safety and anti-tumour immunity of this approach was recently demonstrated in a phase I and a phase II trial (Jaffee *et al*, 2001; Laheru *et al*, 2005).

The purpose of our study was to define the toxicity and efficacy of the toll-like receptor 2/6 agonist MALP-2 (Morr *et al*, 2002) in combination with gemcitabine for the treatment of unresectable pancreas carcinoma. The rationale of this approach is based on the ability of MALP-2 to (1) act as an immunological adjuvant (Rharbaoui *et al*, 2002), (2) activate murine as well as human DC to express co-stimulatory molecules (Weigt *et al*, 2003), (3) induce a T-helper 1 type of response (Wittke *et al*, 1999) and, most importantly, prolong survival in a mouse model of an orthotopic, syngeneic pancreas tumour (Schneider *et al*, 2003). In these experiments MALP-2 was administered by intratumoural injection, alone, or in combination with systemic gemcitabine. We interpreted the beneficial effects were due to immune activation, as we observed an increase in the expression of co-stimulatory molecules on lymphocytes, and cytotoxic T and NK cells infiltrating the tumour. For technical reasons, the pharmacokinetic data were not evaluated in this trial. However, animal experiments in diabetic mice showed disappearance of MALP-2 activity from the skin with a half time of about 20 h (Deiters *et al*, 2004). MALP-2 is degraded by two different mechanism in inflamed tissue: (1) de-esterification and (2) oxidation of the thioester bridge (Mühlradt *et al*, 1998).

## MATERIALS AND METHODS

### Design and setting

Patients who underwent laparotomy with incomplete or no resection of pancreatic adenocarcinoma were treated intraoperatively with 20–30 *μ*g MALP-2. The later was administered intratumourally after careful aspiration to prevent intravasal injection. The maximum injected volume was 750 *μ*l distributed in up to eight single injections, to cover the whole tumour. The dose of MALP-2 was escalated in 5 *μ*g steps after treatment of two patients until CTC>grade 2 was achieved. A postoperative CT with gemcitabine administered in an outpatient setting followed as soon as possible. Gemcitabine was given at a dosage of 1000 mg m^−2^ on days 1, 8, 15, 22, 29, 36, and 43, with a break on day 50 followed by 4-week cycles over 3 weeks of therapy duration. Patients with prior CT and/or radiotherapy (RT) were included in the trial.

### Participants

In phase I/II trial of this study, patients with attempted surgical resection were selected while those with R0 or R1 resections were excluded as there are well-known standard therapies available. In addition, patients with peritoneal carcinosis were not considered, because targeted injection is not possible in disseminated disease. Patients with suspected hypersensitivity to TNF-*α* were excluded as well.

Male and female patients with pancreatic adenocarcinoma were eligible for participation in the study. Patients whose carcinomas were unlikely to be completely resectable upon medical examination were informed about this study. The nature, scope, and possible consequences of the trial were explained by a physician, and informed consent was obtained from each patient in oral and written form before inclusion in the trial. The final protocol was approved by the ethics committee of the University of Heidelberg, Medical School (L-239/2003). The protocol has been published in October 2003 on www.kimt.de with the number HD239. The first patient was enrolled in April 2004.

### MALP-2

The biologically active *R*-stereoisomer of MALP-2 was synthesised and HPLC-purified as described (Morr *et al*, 2002). A stock of 1 mg ml^−1^ MALP-2 solution was prepared in pyrogen-free water containing 33% v v^−1^ 2-propanol, and the exact content was determined by amino acid and fatty acid analysis. MALP-2 solutions for injections were then made up to a final concentration of 40 *μ*l ml^−1^ in water with 10%, v v^−1^ 2-propanol and 2% wt v^−1^ human serum albumin 20%. The biological activity of MALP-2 was tested by nitric oxide release as described elsewhere (Mühlradt *et al*, 1997). Each lot of MALP-2 was tested for sterility at the Department of Hygienic, University of Heidelberg. Endotoxin contamination was examined by two methods: (1) LAL-assay at the Department of Hygienic, University of Heidelberg (2) a very sensitive nitric oxide release assay with TLR-2 knockout mice. Cells from these animals did not react to the MALP, as MALP uses TLR 2/6 as receptor, whereas LPS uses TLR 4. The test would have indicated even small amounts of LPS if it had been present.

### Immunomonitoring

Peripheral blood mononuclear cells (PBMC) were isolated and stored frozen at −80°C. Wound secretion was obtained either directly during the operation or later on from EasyFlow drainage. During the operation, wound secretion was obtained by a syringe inserted close to the injection site. Afterwards, EasyFlow drainage was placed close to the tumour, collected wound secretion was taken from the drainage at the indicated time points and receptacle was emptied. Blood was taken from patients shortly before MALP-2 injection and after 1, 6, and 16 h and 3 days following the injection. Blood and wound secretion samples from untreated patients with pancreatic adenocarcinoma who gave their informed consent were obtained as controls.

Leucocytes were analysed after the lysis of erythrocytes, by flow cytometry on an EPICS XL (Coulter, Krefeld, Germany) as previously described (Schmidt *et al*, 2007). Serum cytokine levels were determined using FlowCytomix technology (Bender Med Systems, Vienna, Austria) (Schmidt *et al*, 2007). The Human Granzyme B ELISpot Set was used according to the manufacturer's instructions (BD, Heidelberg, Germany) with 400 000 PBMCs per well and stimulation with 100 *μ*g ml^−1^ MUC-1, 100 U ml^−1^ CA19.9 (Merck, Schwalbach, Germany), or medium as a control. Spots were counted after 24 h of incubation. Deep-frozen PBL were used for the NK cytotoxicity assay against K562 cells in a standard chromium release assay (Schmidt *et al*, 2007).

### Statistical analysis

Mann–Whitney *U*-test, Pearson's correlation, log-rank test and paired *t*-test on SPSS 11.5 were used to analyse statistical significance where appropriate. A *P*-value <0.05 was considered as significant.

## RESULTS

### Patients

Ten patients, four females and six males, were included in this study and all received MALP-2. Median age was 68.5 years. Five patients were R2 resected, one underwent hepatojejunostomy plus gastrojejunostomy, three hepatojejunostomy, and one underwent explorative laparotomy. Four patients had received neither pre- nor intraoperative RT nor CT, two had undergone IORT, two IORT and RT preoperatively, and two had received IORT and radiochemotherapy (RCT) before surgery, and two had received CT, RCT, and IORT before surgery. They were treated postoperatively with gemcitabine, or received additional RCT. One patient was treated with hyperthermia on his own will (see Table 1). All patients had T3 adenocarcinomas, 50% had positive lymph nodes, and median age was 69 years.

Three patients received no postoperative therapy, one of them died on day 54 because of rapid progression of the disease. Another patient died on day 49 due to postoperative complications, and one patient did not recover from his myocardial infarction 3 days after surgery and was therefore not further treated.

### Toxicity

An MALP-2 dose of 20 *μ*g given to five patients was generally well tolerated. Neither vital signs nor routine laboratory data differed from those of untreated control patients after surgery of pancreatic carcinoma. Serious adverse events (SAE) were recorded in two out of three patients treated with 25 *μ*g MALP-2. Patient 4 with a known history of myocardial infarction suffered a myocardial infarction 3 days following surgery and concomitant MALP-2 injection. A correlation of this SAE with MALP-2 treatment was defined as ‘not assessable’. Patient 5 suffered from three episodes of cardiac arrest 18 h post-intraoperative MALP-2 injection. He was successfully resuscitated, and a temporary cardiac pacemaker was implanted. The patient completely recovered. Patient 6 was treated with 30 *μ*g MALP-2 and 15 min after injection she became hypovolemic and required volume resuscitation and vasopressors. The patient recovered within 2 days. Retrospectively, accidental intravenous injection of MALP-2 could not be rigorously excluded as aspiration was not performed in this patient to explain the early shock-like symptoms. However, CRP levels, that should be indicative of a systemic MALP-2 reaction, were lower than in control patients (see also below). Patient 6 developed an abdominal sepsis with infected ascites 2 weeks after surgery. She died of septic multi-organ failure on day 49. In patient 7, who received 30 *μ*g MALP-2 as well, no side effects were noticed.

### Clinical outcome

**Figure 1:**
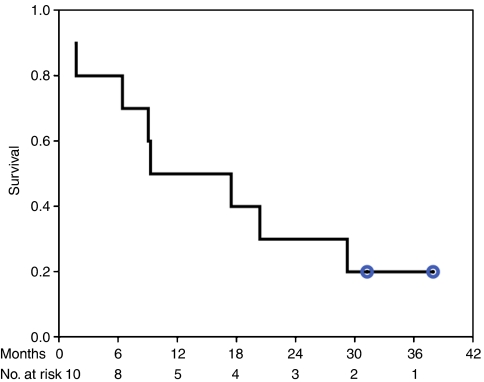
Overall survival of patients. Ten patients were treated with various doses of MALP-2.

Although our study was a phase I/II trial primarily focusing on toxicity, dose escalation and immuno-monitoring, the clinical outcome was a secondary objective. The mean survival is 17.1±4.2 months (CI 8.9; 25.3); the median survival was 9.3 months. Two patients were still alive after 31 months (Figure 1). There were no significant differences in survival rates of patients with regard to the different surgical approaches applied (R2 resection *vs* palliative surgery), intraoperative strategies (IORT *vs* no RT), or the MALP-2 dosage. No metastases were reported during follow-up.

### Laboratory parameters

**Figure 2:**
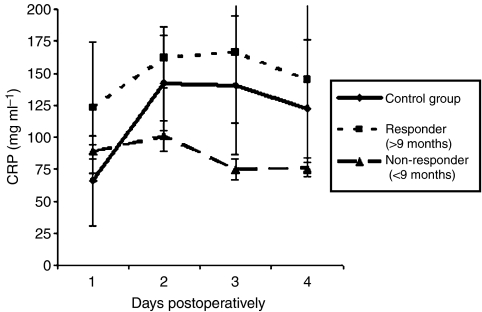
CRP serum level. CRP serum level from MALP-2 patients was monitored during the first 4 days postoperatively. Data from 17 patients underwent pancreatic surgery were taken retrospectively. Responder and non-responder were defined as survival > or <9 months survival, respectively. Data are shown as mean±standard deviation.

The routine laboratory parameters revealed no conspicuous abnormalities, except the CRP values. When we grouped MALP-2-treated patients in terms of survival time into responders (survival>9 months) and non-responders (survival <9 months), an interesting pattern became apparent: during the first 4 days after surgery/MALP-2 treatment, CRP levels in responders were significantly higher than in non-responders (survival <9 months; *P*<0.001) or control patients (*P*<0.035). On the other hand, there was no significant difference between non-responders and control patients (*P*<0.8). After day 5 all groups became identical (Figure 2).

### NK cell–mediated cytotoxicity

**Figure 3:**
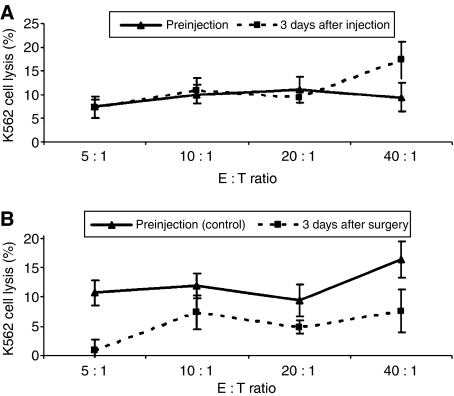
(**A** and **B**) Cytotoxicity of peripheral blood lymphocytes. Peripheral blood mononuclear cells were thawed 1 day before analysis. Monocytes were removed by plastic adherence. Cytotoxic activity was determined in a standard chromium release assay against the classical NK cell target K562. Data are shown as mean from 10 MALP-2 patients (**A**) and 4 control patients (**B**). Data are shown as mean±s.e.m.

Upon monitoring the NK cell activity against K562 targets before and after surgery, the control group, who had not received MALP-2, showed a suppression of NK activity on day 3 as compared to values from samples taken during surgery. This effect was overcome in the MALP-2-treated patients. Thus, NK activity on day 3 was significantly higher in MALP-2 patients than in control patients (*P*<0.03; Figure 3A and B). This observation, when supported by more patient histories, may become of prognostic value.

### Leucocyte subpopulations in PBL and wound secretion

**Figure 4:**
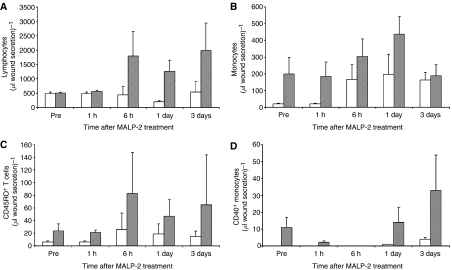
(**A**–**D**) Immunophenotyping of peripheral blood leucocytes and leucocytes in the wound secretion. Leucocytes were analysed immediately after blood drawn. Flow-count fluorospheres were added to samples from wound secretion for counting cells per microlitre. (**A**) number of lymphocytes in wound secretion, (**B**) number of monocytes in wound secretion, (**C**) CD45RO+ lymphocytes in wound secretion, and (**D**) CD40+ monocytes in wound secretion. Data are shown as mean±standard deviation. Control patients are depicted as white bars and MALP-2 patients as grey bars.

Since MALP-2 is known to induce chemokines and, upon local administration, infiltration of leucocytes (Deiters and Mühlradt, 1999), we wished to know whether MALP-2 in the setting of this study could act systemically by influencing the composition of PBL, or whether any effects would be topically restricted and become apparent upon analysis of the wound secretions. Since, there were only small if any effects on the PBL composition, significant changes were seen in the leucocytes from the wound secretion. Lymphocytes as well as monocytes started to increase in number 6 h after MALP-2 treatment. Leucocyte subpopulations in the wound secretion were recorded as percentage as they were heavily contaminated with cells from connective and other tissues. T cells, among them CD45RO+ memory T cells, peaked at 6 h, whereas monocytes showed a maximum value on day 1 and expressed activated CD40+ ones later (Figure 4A–D).

### Granzyme B ELISpot and cytokine profiles

**Figure 5:**
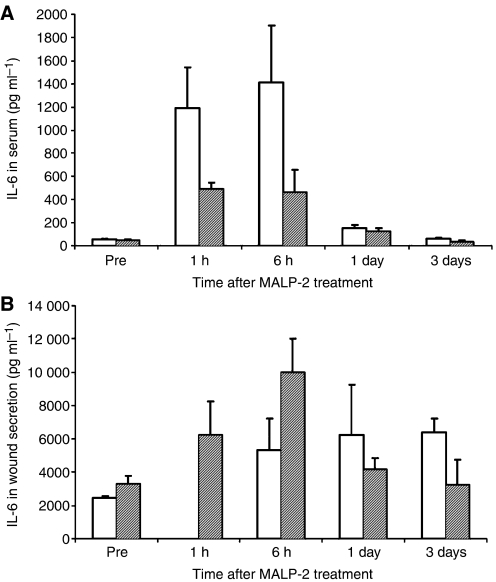
(**A** and **B**) IL-6 levels in the blood and in wound secretion. (**A**) IL-6 level in serum of MALP-2 and control patients. Cytokine levels were determined by FlowCytomix as described. (**B**) IL-6 level in wound secretion of MALP-2 and control patients. Data are shown as mean±standard deviation. Control patients are shown as white bars and MALP-2 patients as grey bars.

Data generated from Granzyme B ELISpot and from cytokine profiling in the serum resulted in no significant difference between control and MALP-2 patients. However, IL-6 levels were high in the wound secretion and in serum and increased with time also in the untreated populations. In MALP-2-treated patients, this effect was more pronounced and appeared earlier (Figure 5A and B).

## DISCUSSION

Carcinomas of the exocrine pancreas have an extremely poor prognosis. Radio- and chemotherapy are not very efficient even after attempted radical surgery. In this respect, combination with immunotherapy could be another strategy for cancer treatment. As pancreatic carcinomas lack well-characterised tumour antigens, unspecific immunotherapy might be a treatment option. Immunomodulators such as chemokines or adjuvants act by inducing cytokine secretion from monocytes or macrophages. They lead to a Th1-skew and cell-mediated immunity. Adjuvants can be the danger signals that are necessary to stimulate DC for optimal antigen presentation and stimulation of effector cells. They may help to link the innate and adaptive immune system.

Tumour-suppressive effects of lipopeptides have been described previously (Fidler *et al*, 1989; Utsugi *et al*, 1991; Xie *et al*, 1995, 1996; Xie and Fidler, 1998; Bruns *et al*, 2000; Galanos *et al*, 2000; Shinohara *et al*, 2000). Recently, we reported the treatment with MALP-2 applied to an orthotopic pancreatic carcinoma mice model. The MALP-2-treated animals showed a significantly prolonged survival, especially in combination with CT. Furthermore, a strong immune response could be observed. Tumours of treated mice were infiltrated with NK and T cells and a Th1 shift was detected. The signal transduction cascade activated by MALP-2 is as well described as the patterns of mediators secreted after injection of the lipopeptide. MALP-2 binds to TLR-2 and TLR-6 on monocytes and other cells. This results in the activation of NF-*κ*B and other transcription factors, as well as the release of pro-inflammatory cytokines (Sacht *et al*, 1998; Deiters and Mühlradt, 1999; Kaufmann *et al*, 1999). Doses up to 1 *μ*g were given s.c. into the wound edge applied by punch biopsy to volunteers undergoing a phase I study about MALP-2 application to heal chronic wounds. Except for erythemas there were no side effects.

After deciding to investigate this approach in a clinical study, we were faced with several obstacles. One of these was related to the order of administering the CT and immunotherapy. Although the commonly used regimen is to start with CT followed by immunotherapy, we could only start with immunotherapy followed by CT due to some technical reasons. On the other hand, we presume that induction of necrosis by surgical intervention also results in the release of immunogens, which could then be taken up by MALP-2-activated antigen-presenting cells.

It is well known that surgery results in immune suppression (Menger and Vollmar, 2004). Furthermore, there is strong evidence from animal data that a whole host of agents that broadly stimulate the immune system are effective in reducing significantly the incidence of tumour metastases and the growth of tumours after surgery. In theory, perioperative immune upregulation would provide the patient with the ability to kill tumour cells immediately following surgery through specific and innate immune responses. We observed an immune-suppressive effect in control patients regarding NK cell–mediated cytotoxicity, which was overcome in MALP-2-treated patients. This might be one explanation for the lowered incidence of metastasis that has been observed in our patients. Indeed, MALP-2 by itself features an anti-metastatic effect in animal experiments (Shingu *et al*, 2003).

The immunomonitoring, as the crucial part of this phase I/II trial, was restricted by the number of specimen we could sample without causing too much inconvenience to our patients and the number of assays we could perform. Since the study investigates an unspecific immunostimulants, and since there are only few potential target antigens described for pancreatic carcinomas, assays like tetramer analysis or peptide-specific ELISpots were not feasible. It was also not possible to establish tumour cell lines from the majority of patients as a source of autologous antigen or as a target in cytotoxicity assays (Keilholz *et al*, 2002). Another general issue in immunomonitoring is the fact that in most cases it is only possible to look for activated cells in the periphery and not in the tumour. We tried to solve these problems by investigating unspecific responses as NK cell mediated cytotoxicity, immunophenotyping, and cytokine serum levels in peripheral blood and in the wound secretion. For the detection of antigen-specific cells we performed ELISpots with whole-tumour antigens (CA 19.9 and MUC-1) which are expressed in tumour cells of >85% of all patients. The time points of blood withdrawal were restricted due to fast-track surgery.

In general, we underestimated the effect of surgical procedures on the immune system and the problems ensued regarding the immunomonitoring. Here, the presence of unaffected NK cell-mediated cytotoxicity in MALP-2 patients is to strengthen. Moreover, an increase in memory T cells and CD40 expression on monocytes, particularly in the wound secretion, was detected. No significant changes in the PBL were noted. Based on this observation, it seems that MALP-2 effects are predominantly local. Unfortunately, we were not able to draw samples in a long enough period of time after intervention (>14 days) for an adequate detection of antigen-specific T cells.

In addition, the patterns of CRP, IL-6, and TNF-*α* levels were unpredicted. CRP is produced almost exclusively by liver hepatocytes as part of the acute-phase response to IL-6, TNF-*α*, and IL-1*β* originating at the site of inflammation. Preoperative and postoperative CRP levels of >10 mg l^−1^ are independent indicators predictive of poor prognosis of patients with pancreatic carcinoma (Jamieson *et al*, 2005). In contrast, our observations—although limited—suggest that patients with a high increase of CRP during the first days had a better outcome than those with the normal postoperative course. The absence of a change in IL-6 and TNF-*α* levels in serum of MALP-2-treated patients was unexpected since control patients peaked within the first postoperative days. One possible explanation could be a targeted, local inflammation in MALP-2 patients that was not detectable in serum or wound secretion resulting in a noticeable CRP increase. In control patients we had an untargeted, surgical procedure-related inflammation, which is measurable in terms of IL-6 and TNF-*α* in serum and wound secretion and resulting also in CRP increase.

The following model might explain our observations: (1) MALP-2 induces local inflammation; (2) surgery and local TNF release by MALP-2 activated monocytes/macrophages and cause tumour destruction; (3) leucocytes, including NK cells are directed to the injection site by the release of chemokines; (4) anergic DC are activated by MALP-2 to mature (Weigt *et al*, 2003). Such DC could take up apoptotic or necrotic tumour cells and prime lymphocytes; (5) these lymphocytes can infiltrate the tumour, as shown in the animal model (infiltration with NK cells and T cells); and (6) MALP-2 prevents metastases (Shingu *et al*, 2003, unpublished data).

Although the cohort is too small for any final conclusions, it seems that the patients had a benefit from the regimen. Pre-, intra- and postoperative therapies are not comparable between the patients but the data did not give the impression that any particular treatment was responsible for a better outcome. The mean survival of 17.1±4.2 months (CI 8.9; 25.3) was surprisingly high. Interestingly, there were no significant differences either between patients who had an R2 resection (R0–R1 resection intended) or palliative surgery, with or without IORT, nor between groups treated with variant MALP doses. Since no toxicity was observed in the patients group treated with up to 20 *μ*g MALP-2, we recommend to use this dosage in further trials.

In conclusion, MALP-2 in dosages up to 20 *μ*g is not associated with side effects. Although the immunosuppressive impact of invasive manipulations compromise immunomonitoring, some interesting activating and anti-immunosuppressive effects were detected. The clinical outcome with a mean survival of 17.1 months is remarkably high. Further studies are required to evaluate the clinical and immunological impact of MALP-2.

## Figures and Tables

**Table 1:** Patients' Characteristics

	**Age**	**Gender**	**Dosage (*μ*g)**	**Type of operation**	**Preoperative therapy**	**Postoperative therapy**	**Side effects (CTC grade)/relationship**	**OS (months)**	**Status**
1	63	M	20	R2	CT, RCT, IORT	CT	—	38.0	Alive
7	67	M	30	Palliative	IORT	RCT	—	31.3	Alive
5	63	M	25	Palliative	CT, RCT, IORT	CT	Asystole (3), possible	29.3	Died
3	74	M	20	R2	—	CT	—	20.4	Died
8	69	F	20	Palliative	IORT	CT	—	17.4	Died
10	68	M	20	Palliative	—	Hyper-thermia	—	9.3	Died
2	37	F	20	R2	—	CT	—	9.0	Died
4	73	M	25	R2	RCT, IORT	—	Myocardial infarction (4), not assessable	6.5	Died
9	69	F	25	Palliative	—	—	—	1.8	Died
6	69	F	30	R2	RCT, IORT	—	Endotoxin-like shock (4), probably	1.6	Died

Abbreviations: CT, chemotherapy; CTC, common toxicity criteria; F, female; IORT, intraoperative radiotherapy; M, male; OS, overall survival; RCT, radiochemotherapy.
